# A comprehensive review of the components of nurse-coordinated care which are most effective in preventing coronary heart diseases

**DOI:** 10.4314/ahs.v23i1.55

**Published:** 2023-03

**Authors:** Wenna Fan, Chao Guo, Qi Zhao, Hongbo Ma

**Affiliations:** Outpatient pediatrics, The 2^nd^ Affiliated Hospital of Harbin Medical University, Harbin City, Heilongjiang Province, China

**Keywords:** Coronary Artery Disease (CAD), Nurse-Coordinated Care (NCC), Nursing Interventions (NI)

## Abstract

Coronary artery disease occurs when there is inadequate blood flow to the heart muscle as a consequence of coronary artery blockage, resulting in heart muscle failure. During normal heart action, cardiac muscles will always need an adequate supply of blood to fulfill their oxygen requirements. Coronary heart disease is the most common kind of cardiovascular disease in adults and the leading cause of mortality in the United States. Growing understanding of the possible significance of environmental and lifestyle variables in disease development has enhanced the job of the nurse coordinator, whether at a lower or higher level of responsibility, to keep current ondiagnostic procedures, clinical symptoms, and innovative treatment choices. According to the national cardiovascular control program, secondary prevention of cardiovascular disease has increased, including measures such as cholesterol management, blood pressure monitoring, and smoking cessation. If you know more about NCC, it might be easier to figure out what roles it could play and what effects its use might have.

## Introduction

Heart disease (CAD) is the main cause of morbidity and mortality on a global scale, and it accounts for a higher proportion of total economic expenditures than any other kind of illness. The first sign of cardiovascular disease is when fatty substances and fibrous structures build up in the coronary arteries, which are the blood vessels in the heart.^1^ The accumulation of lipids over time causes the narrowing of blood channel lumens, which culminates in the creation of myocardial layers in the heart. Once lipids have accumulated, they continue to damage artery walls via structural and metabolic changes, which result in physiological changes as a consequence of these changes.^2^ Acute coronary syndrome (ACS), the most severe symptom of coronary artery disease (CAD), results in immediate hospitalization.^3^,^4^ Traditional risk factors for coronary artery disease include advanced age, gender, and a family history of coronary artery disease, as well as unhealthy lifestyle factors like being overweight, smoking, and stress, and comorbidities like high blood pressure, high cholesterol, and diabetes.^5^,^6^ Traditional risk factors are thought to be responsible for around 75% of all cases of coronary artery disease. The kind of coronary artery blockage or damage determines the type of treatment for coronary artery disease in the modern day. Some treatments include percutaneous coronary interventions (PCIs) (coronary stents and balloon angioplasty), coronary artery bypass grafting (CABG), and drug therapy.^7^ Attending cardiac rehabilitation programmes has also been shown to be beneficial to patients in terms of improving their overall quality of life.^8^ Because of these changes in medical technology, the nurse's important job has also changed a lot.

## Nurse's role (Nurses' roles and impact in caring for CAD patients)

• Since the 1970s, nurses have been an important part of treating people with one or more coronary artery disease (CAD) risk factors. This has been accomplished via the establishment of specialised clinics and programmes in primary care settings, enterprises, and cardiac rehab institutions.^9^ Nursing duties for CAD patients include education on advanced treatments for the treatment of coronary artery disease (CAD), nutrition and lifestyle counselling for risk factor management, and development of cardiac rehabilitation skills.^10^

• According to a systematic review of randomised controlled trials, nurse interventions (NI) for patients with coronary artery disease (CAD) improve blood pressure (BP), lipids, physical activity, food intake, cigarette smoking, weight loss, healthcare utilisation, mortality, quality of life, and psychosocial outcomes in patients with CAD. On the other hand, more than half (65%) of the treatments used in the study were education and behavioural counselling. On top of being different, some studies couldn't figure out which part of the intervention caused the change in outcome.^11^

• Another review of randomised controlled trials done in residential and outpatient settings found that coordinated cardiovascular nursing care (CVNC) had a statistically significant effect on lowering blood pressure in patients with coronary artery disease.^12^

• There was heterogeneity in the data, according to the results of the most recent systematic review and meta- analysis on the components of nurse-coordinated care that are most useful in avoiding recurrent cardiac arrhythmias.^13^ The authors, on the other hand, used a descriptive method in order to summarise the various NI components and their impact on outcomes. Their consensus resulted in the agreement on three unique intervention possibilities, which were as follows: The following are the steps involved in risk factor management: responsibilities include: (1) prescribing and/or titrating medications, educating patients about risk factors, counselling patients on diet and lifestyle modification, monitoring vital signs such as blood pressure, cholesterol control, and detecting and counselling for stress/depressed mood; (2) multidisciplinary consultation, including referral; and (3) shared decision-making, which includes goal setting for an individualised self-care plan and family support Additional important research questions about the feasibility of employing the suggested therapeutic approaches within the context of clinical practise were raised, taking into consideration the fact that statistically significant findings are not always predictive of clinically meaningful findings in all circumstances.^14^

According to the conclusions of a study on nurses' participation in cardiovascular risk assessment and communication, there are seven essential themes for nurses' role in CVD risk assessment and management.

1. Incorporation of WHO/ISH charts into standard nursing procedures

2. Patients are not being communicated with.

3. The passage of time

4. A lack of performance evaluations and evaluations based on performance.

5. The scope of risk shifting is being expanded.

6. Infrastructure is number six.

7. Nurses' education and training facilities are number seven.

8. Team members' assistance

• Nurses' guided care management has been shown to be very beneficial in lowering risk factors and treating both young and older people with co-morbidities, as well as treating the general population.^15^ Nurses' effectiveness in trying to modify a broad population with various risk factors is mostly dependent on educating and counselling those who are not motivated to change their behaviour.

• The researchers discovered a decrease in mortality and acute MI in a meta-analysis of secondary CVD prevention programmes, which was conducted by Clarks and colleagues. The researchers discovered that 45% of the trials included in the study were either directed by nurses or supervised by nurses.^16^ It was proven in a 2016, cardiac hospitalization atherosclerosis management programme on the use of guidelines-based therapy for CVD risk reduction that patients who received nurse-directed care management saw a decrease in both mortality and morbidity.^17^

• There are more than 10 million nurses in the globe who represent the health-care provider team and who have the necessary education and position to play a role in the management of CVD risk reduction in the population.^18^

• A research that examined the effect of community-based nursing interventions in improving outcomes for persons with cardiovascular disease revealed that they did so in four critical areas, including self-care, health, healthcare use, and quality of care. This also revealed that the nurses' lack of time and expertise was a significant obstacle to intervention effectiveness. The project, titled “Task shifting of cardiovascular risk assessment and communication by nurses for primary and secondary prevention of cardiovascular illnesses in a tertiary health care context in Northern India,” is scheduled to conclude in 2020. When nurses' risk assessments were compared to the investigator's, a significant degree of agreement (k = 0.84) was observed.^19^ When compared to the baseline evaluation, the primary preventive group had a considerably larger number of participants (70%) in the low-risk category at the one-year follow-up (60.6%). The mean medication adherence score (7.60) was significantly higher in the intervention group than in the comparison group (5.96) in the secondary prevention group, with a large effect size of 1.1 (p 0.05 and/or 0.01) and an adjusted odds ratio (95 percent confidence interval) that were within their statistically significant range. Diabetes mellitus patients were five times more likely than the rest of patients with coronary artery risk factors to exert influence on the delivery of “administer drugs and their instructions” (p 0.01, AOR (95 percent confidence interval) 5.02).^20^

• According to 2011 research, the global cost of circulatory system illnesses is considerable on a personal and social level. International research efforts have concentrated on discovering the most effective and cost-effective methods to adopt preventative interventions. When preventative programmes were implemented by teams of health care professionals with competence in nursing, dietetics, physical activity, and behavioural skills, they were especially successful in high-risk and vulnerable groups. When implemented properly, team-based and nurse-directed case management has the potential to have a significant influence on both primary and secondary prevention of cardiovascular and other vascular illnesses.^21^

A 2018 article discusses how coronary artery disease (CAD) is one of the most common cardiovascular diseases afflicting the global population and how it may be prevented. Both developed and developing countries have been demonstrated to be afflicted by this disease, which is the main cause of death in both. Coronary artery disease is a result of a mix of lifestyle choices, environmental influences, and genetic predispositions. The predominance of risk variables in healthy individuals indicates that coronary artery disease (CAD) will almost certainly develop shortly. According to genome-wide association analysis, a link has been shown between chromosome 9p21.3 and the beginning of coronary artery disease (CAD) at a young age. Diabetes, hypertension, tobacco use, hyperlipidemia, obesity, homocystinuria, and psychological stress all contribute to an increased risk of coronary artery disease (CAD). Numerous investigations and clinical trials have shown the effectiveness of CAD removal and therapy. Antiplatelet agents, nitrates, beta-blockers, calcium and antagonists are only a few of the few current therapeutic options for symptomatic angina associated with coronary artery disease.^22^

• Cardiovascular disease (CVD) is the leading cause of morbidity and early death in both men and women across the globe. Approximately 12 million nurses work in the world's biggest health-care profession, which is responsible for treating cardiovascular disease risk factors and chronic illnesses. It is estimated that nurses and other team members will contribute significantly to achieving the worldwide aim of a 29 percent reduction in cardiovascular disease (CVD) mortality and disability by 2028, as stated by the American Heart Association (AHA) and the World Health Organization (WHO). Nurses and advanced practise nurses have been in charge of treating single and multiple risk factors such as hypertension, smoking, hyperlipidaemiaand diabetes, as well as the squealed of chronic illnesses, for more than four decades now.^23^

• Cardiovascular disease (CVD) is the leading cause of mortality in the United States (source: CDC). Despite the fact that the causes of CVD are many and include both inherited and environmental factors, the illness is mostly preventable. One of the most important aspects of CVD prevention is the capacity to properly predict risk in order to identify people who may benefit from risk-reducing interventions. A large number of nurse practitioners do cardiovascular risk assessments, putting them in a unique position to influence preventive therapy.^24^ Because a family history of early CVD is not considered a major risk factor in current methods for scoring cardiovascular disease risk, the chance of getting the disease is greatly underrated.

• According to current guidelines, nurse-coordinated care (NCC) is an effective technique for secondary prevention of cardiovascular disease. However, NCC programmes differ significantly from one another, and the value of NCC components has not been well researched too far. A systematic review and meta-analysis of randomised controlled trials were conducted in order to establish the efficacy of NCC and its components in the secondary prevention of coronary heart disease. There were approximately 3000 randomised trials with 11,195 patients and 18 components of NCC that met the stated inclusion criteria, with a total of 11,195 people. With the use of these components, three unique intervention strategies were developed: Prevention and control of risk factors (15 studies), multidisciplinary consultation (12 studies), and collaborative decision-making are all explored (13 studies). Six studies used parts of all three NCC approaches into their designs. There was a total of 35 outcomes that were observed. The observed findings were divided into four groups, which are as follows: 1-The concentrations of risk factors (18 studies) 2-Clinical occurrences (9 studies); 3-Patient's perception of his or her own health (9 studies) 4-Adherence to established procedures (5 studies). When compared to conventional care, NCC substantially lowered systolic blood pressure (weighted mean difference (WMD) 2.96 mm Hg; 95 percent confidence interval (CI) 1.53 to 4.40 mm Hg) and low-density lipoprotein cholesterol (WMD 0.23 mmol/L; 95 percent CI 0.10 to 0.36 mmol/L). In addition, the National Cancer Institute raised smoking cessation rates by 25 percent. (Risk ratio: 1.25; 95 percent confidence interval: 1.08 to 1.43) NCC has been proven to have a minimal impact on a small number of outcomes over a short period of time. The National Cardiovascular Control Program (NCC), which includes blood pressure monitoring, cholesterol treatment, and smoking cessation, had an impact on improvement in secondary prevention. Besides that, NCC is an ambiguous concept. NCC should be defined consistently in order to allow for more precise comparisons of its content and repercussions to be made.

On the basis of the Galway Consensus, Virna Ribeiro Feitosa CestariI and colleagues released a review with the goal of identifying nurse competencies important to chronic heart disease health promotion in the year 2016. They observed, nursing interventions for chronic cardiac patients' health promotion used all areas of competence, with Planning and Evaluation being the most prevalent. The results of this research established the nurse as a competent agent capable of managing care in order to improve the coordination of the latter with employment and education, and therefore with the health-care needs of the general population.^26^

In order to establish the efficacy of community-based nursing treatments in improving outcomes for community-dwelling adults with cardiovascular disease risk factors, a study was conducted (CVD) There were 46 studies that met the criteria for inclusion.^27^ Symptom control, symptom awareness, symptom management, and social outcomes have all been shown to be improved with community nursing interventions. Treatments that are beneficial have been shown to be facilitated by a community-centric strategy, participant empowerment, reinforcement mechanisms, a targeted approach to poor populations, and home visits In patients with hyperlipidaemia, these therapies resulted to significant reductions in HbA1c levels in diabetic patients, accomplishment of blood pressure goals in hypertensive patients, and a significant improvement in self-reported food intake in patients with diabetes, among other benefits.

An investigation on “The Role of Nursing Education Following a Cardiovascular Event” was conducted. Individuals who have had a big cardiovascular event may be concerned about a range of problems related to the disease's prognosis or rehabilitation, as well as anxieties about returning to their previous lifestyle. Recovering after a cardiac event is a time-consuming process that comprises both psychological and physical needs that continue after the patient has been discharged from the hospital. With this study, we hoped to better understand the significance of post-cardiac event or procedure nursing education.^28^

Components and outcomes: A large number of studies have shown the crucial role played by nurses in cardiac rehabilitation programmes. According to the available data, a nurse-led instruction programme is associated with a decreased incidence of difficulties, anxiety, and readmissions to the hospital after cardiac events. Additional research shows that incorporating therapeutic lifestyle modifications into a nursing programme may significantly reduce cardiac risk factors and, in some cases, improve prognosis.^29^

## Health literacy and coronary heart disease

Health literacy is a problem that may have a significant influence on patients who are enrolled in self-management programmes that involve drug adherence as well as lifestyle modifications. In addition, patients with lower levels of health literacy ask fewer questions and, as a result, are more likely to make mistakes. This has the potential to have serious effects for the patient, his or her family, and health-care professionals.^30^ This article provides a review of the key issues related to health literacy, including how it is defined; the impact of health literacy on health outcomes, why it is a useful concept for cardiac nurses to be aware of, and strategies cardiac nurses should consider to improve patients' health literacy. It is written for cardiac nurses who want to learn more about health literacy.31 It is well established that people's perceptions of their own health are influenced by their degree of health literacy. Poor health literacy has an impact on people from all walks of life. The suggestions provided here might help cardiac nurses improve their educational efforts, resulting in each client being more health-literate and efficient in their own self-care.

## Conclusion

CAD has been identified as a significant source of death and morbidity in the human population, and it has also been identified as being related with a variety of foetal co-morbid conditions. Apart from lifestyle and environment, there are numerous other factors that contribute to the development of the disease. Genetics plays a significant role in the lack of education among the general public regarding the early detection and identification of risk factors for coronary artery disease (CAD). Nurses play an important role in the education, treatment, and management of coronary artery disease (CAD). Increasing the use of evidence-based nursing has been demonstrated to be useful in the prevention of CAD.

## Figures and Tables

**Figure 1:**
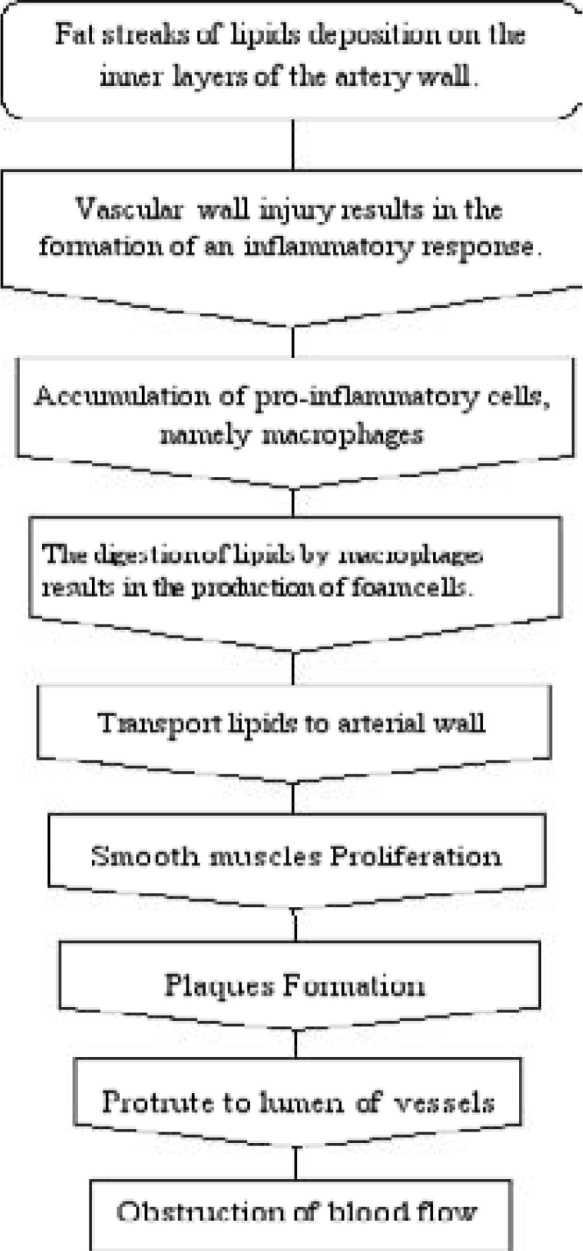
Cardiovascular Disease Pathophysiology.
